# Tracking emotions and emotion regulation strategies used in a hospital staff cohort during the COVID-19 pandemic

**DOI:** 10.1192/j.eurpsy.2021.781

**Published:** 2021-08-13

**Authors:** A. Goldring, B. Silston, S. Krueger, J. Bendt, M. Loguidice, N. Wimberger

**Affiliations:** 1 Psychiatric Research, Nathan Kline Institute, New York, United States of America; 2 Psychology, Columbia University, New York, United States of America; 3 Basic Science, The Ohio State University, Columbus, United States of America; 4 Psychiatry, Manhattan Psychiatric Center, New York, United States of America

**Keywords:** stress, emotion regulation

## Abstract

**Introduction:**

In response to collective life events, many people regulate their emotional states through social interactions to reduce cognitive tolls. During pandemics, physical distancing renders the social support strategy less viable, increasing mental health risks.

**Objectives:**

The current work aims to understand the range of emotions and strategies used in a population of Mental Health staff.

**Methods:**

We conducted an anonymous survey on staff from OMH facilities (n=211) to assess the impact of Covid-19. The current survey captures a host of social, affective, and demographic variables. Accompanied by scales on emotions, emotion regulation, risk, and perception.

**Results:**

Work, family, and health-concerns were the primary contributors to mood. The most common strategy was “situation-avoidance,” then “exercising.” When comparing depression scores against whether or not specific kinds of regulation strategies were utilized, only differences in the strategy of “emotion-suppression” and “authority-seeking” were substantially related to the CES-D scores. Specifically, participants who kept feelings to themselves tended to have higher CES-D scores than those who let their feelings show, while those who sought out authority scored lower on the CES-D, on average than those that did not seek authority.

**Conclusions:**

Healthcare staff are under greater stress and pressure during national emergencies, and to regulate emotions during consistent high-stress, our preliminary data suggest that suppression and distraction strategies are dominant. Suggesting that staff prefer or have little choice but to focus on work. Alternatively, perhaps they are overwhelmed to the extent that avoidance and distraction are more accessible strategies. Further analysis of our data may help us understand more.

